# Novel Mode Engineering for β-Alanine Production in *Escherichia coli* with the Guide of Adaptive Laboratory Evolution

**DOI:** 10.3390/microorganisms9030600

**Published:** 2021-03-15

**Authors:** Jian Xu, Li Zhou, Meng Yin, Zhemin Zhou

**Affiliations:** 1 The Key Laboratory of Industrial Biotechnology of Ministry of Education, School of Biotechnology, Jiangnan University, 1800 Lihu Avenue, Wuxi 214122, China; xujiane_mail@163.com (J.X.); lizhou@jiangnan.edu.cn (L.Z.); 6200208147@stu.jiangnan.edu.cn (M.Y.); 2 Food Biotechnology Research Institute, Jiangnan University (Rugao), Rugao 226500, China

**Keywords:** anaerobic fermentation, energy regulation, CO_2_ fixation, adaptive laboratory evolution, β-alanine production

## Abstract

The strategy of anaerobic biosynthesis of β-alanine by *Escherichia coli* (*E. coli*) has been reported. However, the low energy production under anaerobic condition limited cell growth and then affected the production efficiency of β-alanine. Here, the adaptive laboratory evolution was carried out to improve energy production of *E. coli* lacking phosphoenolpyruvate carboxylase under anaerobic condition. Five mutants were isolated and analyzed. Sequence analysis showed that most of the consistent genetic mutations among the mutants were related with pyruvate accumulation, indicating that pyruvate accumulation enabled the growth of the lethal parent. It is possible that the accumulated pyruvate provides sufficient precursors for energy generation and CO_2_ fixing reaction catalyzed by phosphoenolpyruvate carboxykinase. B0016-100BB (B0016-090BB, *recE*::FRT, *mhpF*::FRT, *ykgF*::FRT, *mhpB*:: *mhpB* *, *mhpD*:: *mhpD* *, *rcsA*:: *rcsA* *) was engineered based on the analysis of the genetic mutations among the mutants for the biosynthesis of β-alanine. Along with the recruitment of glycerol as the sole carbon source, 1.07 g/L β-alanine was generated by B0016-200BB (B0016-100BB, *aspA*::FRT) harboring pET24a-*panD*-*AspDH*, which was used for overexpression of two key enzymes in β-alanine fermentation process. Compared with the starting strain, which can hardly generate β-alanine under anaerobic condition, the production efficiency of β-alanine of the engineered cell factory was significantly improved.

## 1. Introduction

β-Alanine is widely used as an important precursor for the production of pharmaceuticals, chemicals, and foodstuffs. Thus, it boasts high potential in market demand and a sound prospect for development [1]. Traditionally, β-alanine is produced from chemical synthesis, which is a violent chemical reaction with a mixture of acrylonitrile and ammonia. This chemical synthesis process is known to be hazardous to the environment. Moreover, it may also lead to an undesirable pathway to producing polymeric structures [2], which may unintentionally reduce the substrate conversion. Keeping up with the developing trend, more studies have been carried out to investigate more economical and sustainable methods for the industrial production of β-alanine [1,2].

**Figure 1:**
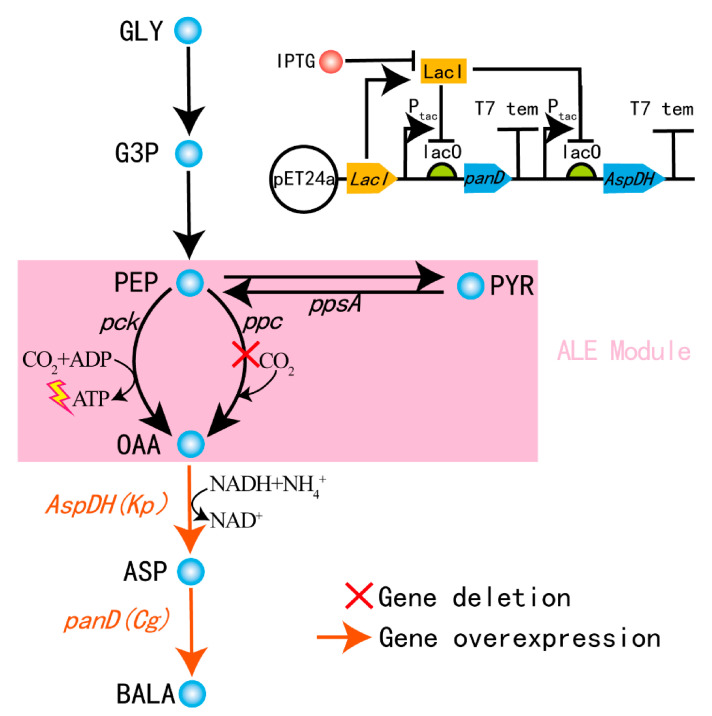
The metabolic pathway designed for the production of β-alanine. The structure of the expression vector pET24a-*panD*-*AspDH* is shown beside the metabolic pathway. GLY: glycerol, G3P: glyceraldehyde-3-P, PEP: phosphoenolpyruvic acid, PYR: pyruvate, OAA: oxaloacetic acid, ASP: aspartic acid, BALA: β-alanine.

For β-alanine biosynthesis, three candidate metabolic pathways have been reported, and the reductive branch of the TCA cycle under anaerobic processes is considered to be the most profitable and greener method [3]. In this metabolic pathway, shown in Figure 1, CO_2_ can be captured and converted into oxaloacetate (OAA), which is related to the biosynthesis of β-alanine. Researchers have focused on this aspect; however, tremendous challenges exist in anaerobic fermentation caused by the lack of sustainable energy supplication, leading to weaknesses in cell growth and proliferation. Both cell growth and β-alanine synthesis are energy-consuming processes. Because of this challenge, the productivity of β-alanine was limited [3].

Adenosine triphosphate (ATP) is known as the most important energy source for metabolic reactions and plays an important role in cell growth and target metabolite production [4]. Therefore, the manipulation of ATP supply could be a powerful tool to satisfy the great demand for energy in the anaerobic fermentation of β-alanine synthesis [5]. Examining the overall metabolic pathways, oxidative phosphorylation and substrate-level phosphorylation are the two main ways to regulate ATP in microorganisms, with the first being more efficient [6]. Under the circumstances of anaerobic fermentation, however, the process of the oxidative phosphorylation pathway is limited by the oxygen supplication, and substrate-level phosphorylation is the only way to regulate the intracellular ATP concentration. Therefore, the metabolic pathway of substrate-level phosphorylation should be reinforced and serve as the source of energy in the biosynthesis of β-alanine.

The production of β-alanine from glycerol is primarily determined by carbon partitioning at the phosphoenolpyruvate (PEP) node. Both endogenous enzymes of phosphoenolpyruvate carboxylase (PPC, encoded by *ppc*) and phosphoenolpyruvate carboxykinase (PCK, encoded by *pck*) can catalyze the carboxylation of PEP to oxaloacetate [7], and then OAA is further converted to β-alanine catalyzed by aspartate dehydrogenase (AspDH, encoded by *AspDH*) and L-aspartate-a-decarboxylase (ADC, encoded by *panD*). To investigate carbon partitioning and energy regulation, great attention should be paid to the reaction from PEP to OAA. Moreover, in this node, CO_2_ can be taken into cells and fixated by PPC or PCK and subsequently converted into OAA, which is related to the biosynthesis of β-alanine. There are great differences in this biochemical reaction catalyzed by the two enzymes. The first one is enzymatic affinity. The Km for bicarbonate of PPC is 130-fold lower than that of PCK [8,9], which indicates that PPC is more sensitive to the concentration of bicarbonate, such that PPC can catalyze PEP even at a low concentration of CO_2_, while PCK will perform PEP carboxykinase at a high concentration of CO_2_. The second difference is energy regulation. One stoichiometric ATP can be generated from PEP to OAA catalyzed by PCK rather than PPC [10]. Owing to the great demand for energy in the anaerobic fermentation of β-alanine synthesis, we prioritized using PCK to biosynthesize β-alanine by fixing CO_2_, which can not only reduce the net release of CO_2_ into the atmosphere, but also convert CO_2_ into value-added multicarbon molecules by engineered biological systems [11]. However, the PPC-deficient mutant is lethal for the breakoff of the metabolic flux at the PEP node [12], and experiments should be carried out for the anaplerosis of OAA based on elaborate metabolic regulation strategies.

Adaptive laboratory evolution (ALE) is an effective tool for both improving microbial phenotypes and investigating biological phenomena [13]. Experiments are carried out in a chosen environment that will enhance the selection of mutants and naturally select beneficial mutants, which gain the ability to fit the changed culture conditions frequently and also be long lasting [14]. The main work of synthetic biology is to design metabolic pathways to reprogram cell behavior, as expected, for practical applications such as growth rate and production of target products. Rational design is regarded as a powerful tool to engineer and construct metabolic pathways. However, this strategy requires a sufficient knowledge base and tremendous workload. ALE is considered to be the replenishment strategy of rational design that discovers genome-wide mutations from fitness advantages and then selects and screens the adapted mutants and explains the mechanisms [15]. For the purpose of improving the supply of energy in the lethal strain B0016-090BB (B0016-082BB, *ppc*::FRT), ALE is recruited to enhance the growth of the PPC-deficient mutant to recover the supply of OAA while catalyzing PEP to OAA by PCK and fill the gap of this neglected research area.

The bioconversion catalyzed by the enzyme AspDH from OAA to L-aspartate is a reduction power-dependent process, the rationing of NADH can facilitate the production of β-alanine, and the most efficient and direct NADH source is substrate fermentation. Compared with glucose, a food-carbon source, glycerol is a more reductive carbon source [16], which generates 2 mol NADH in the Embden–Meyerhof–Parnas (EMP) pathway and converts 1 mol glycerol to 2 mol pyruvate. Therefore, the utilize of glycerol could increase the yield of β-alanine.

In this study, *E. coli* was metabolically engineered for the production of β-alanine with the guidance of ALE. Five mutants were isolated and analyzed. Sequence analysis showed that all of the consistent genetic mutations among the mutants were related to pyruvate accumulation, indicating that pyruvate accumulation enabled the growth of the lethal parent. B0016-200BB (B0016-100BB, *aspA*::FRT) was engineered based on the analysis of the genetic mutations among the mutants for the production of β-alanine. Along with the recruitment of glycerol as the sole carbon source, 1.07 g/L β-alanine was generated by B0016-200BB harboring pET24a-*panD*-*AspDH*, which used for overexpression of two key enzymes in β-alanine fermentation process. Compared with the wild type, which can hardly generate β-alanine under anaerobic condition, the production efficiency of β-alanine of the engineered cell factory was significantly improved.

## 2. Materials and Methods

### 2.1. Strains and Plasmids

The genotypes of the microbial strains and plasmids used in this study are listed in Table 1. *E. coli* strain B0016-082BB (B0016-080BB, *panD*:: Cg*panD*) with deletions in various byproduct pathways was reported in our previous study and was recruited and engineered based on the strategy of Red recombination for the production of β-alanine herein. The relevant primers used in the present study are listed in Table S2. The *panD* and *AspDH* genes were codon-optimized based on the *E. coli* code preference and overexpressed on plasmids. The T7 promoter on pET24a was replaced with the tac promoter to generate the pET24a-tac plasmid. The T7 tag and 6 × His tag were replaced with the *panD* gene from *Corynebacterium glutamicum* or the *AspDH* gene from *Klebsiella pneumonia* separately using the ClonExpressI One Step Cloning Kit (Vazyme Biotech, Nanjing, China) to produce plasmids pET24a-*panD* and pET24a-*AspDH*. The cassette of the tac promoter-lac operator-*panD*-T7 terminator was cloned into the plasmid pET24a-*AspDH* using the ClonExpressI One Step Cloning Kit to form the pET24a-*panD*-*AspDH* plasmid. All plasmids used and constructed are listed in Table S1.

### 2.2. Media and Culture Conditions

For gene manipulation and plasmid construction, *E. coli* was cultured in LB medium at 37 °C. The cells were cultured overnight in 50 mL seed medium contained in 250 mL flasks at 37 °C and 200 rpm and then centrifuged at 12,000 rpm for 2 min at 4 °C and subsequently suspended in M9Y medium. The cell suspension was inoculated into M9Y medium to reach an initial *OD*_600_ of 0.05. Cultures were incubated at 37 °C and 200 rpm up to an *OD*_600_ of 6, followed by the addition of isopropyl-1-β-D-thiogalactopyranoside (IPTG) to a final concentration of 0.1 mM to induce gene expression for another 2 h. Then, the cells were anaerobically cultured at 37 °C and 50 rpm. The cell concentration (*OD*600) and the production of β-alanine and L-aspartic acid were periodically monitored. All the experiments were performed in triplicate.

Fed-batch fermentation was carried out in a 5 L bioreactor with 2 L M9Y medium. Cells were precultured up to an *OD*_600_ of 4 and inoculated into the bioreactor (5%, *v*/*v*) for aerobic fermentation. The dissolved oxygen (DO) was maintained above 45% by varying the agitation speed from 200 to 900 rpm and air flow from 2 to 10 L/min. While the cell concentration reached an *OD*_600_ of 70, IPTG was added to a concentration of 0.1 mM to trigger an OFF to ON state for gene expression for another 2 h. Subsequently, anaerobic fermentation was performed with an agitation speed of 50 rpm and without injecting air. The pH was maintained at 7 by automatically feeding 100 g/L NaHCO_3_. A mixture of 650 g/L glycerol, 200 g/L (NH_4_)_2_SO_4_, 4 g/L yeast extract, and 4 g/L tryptone was automatically added into the tank according to the cell growth rate (μ, h^−1^) and specific glycerol consumption rate (qGly, h^−1^) [17].

LB medium contained (per L) 10 g of tryptone, 5 g of yeast extract, and 5 g of NaCl. M9 medium contained (per L) 6 g of Na_2_HPO_4_, 3 g of KH_2_PO_4_, 1 g of NH_4_Cl, 0.5 g of NaCl, 13.21 g of (NH_4_)_2_SO_4_, 5 g of glycerol, and 5 mL of trace metal solution. M9Y media was prepared by supplementing M9 with 2 g/L yeast extract.

### 2.3. Adaptive Laboratory Evolution

#### 2.3.1. Continuous Adaptive Laboratory Evolution

The continuous adaptive laboratory evolution (CALE) experiment was carried out in 250 mL shake flasks containing 50 mL of M9 medium at 37 °C and 200 rpm. The biomass was measured and transferred to fresh M9 medium until the *OD*_600_ was higher than 2. Microorganisms in M9 medium were withdrawn and isolated on M9 agar plates (2% agar powder, *w*/*v*) to select the mutants with the fastest growth rate, and sample were stored at −80 °C for future analysis and as the parent of the next round of CALE experiments performed monthly. When there were no great differences between the mutants and the initial parent strain in terms of growth rate, the expected mutants were achieved.

#### 2.3.2. Marginal Effect-Assisted Adaptive Laboratory Evolution

The marginal effect-assisted adaptive laboratory evolution (MEALE) experiment was first carried out in 250 mL shake flasks containing 50 mL M9Y medium at 37 °C and 200 rpm. The biomass was measured and transferred into fresh M9Y medium until the cell density reached an *OD*_600_ of 2. Microbes in M9Y medium were withdrawn and isolated on M9Y agar plates (2% agar powder, *w*/*v*) to screen for the fastest growing mutants and stored at −80 °C for future analysis; they were used as the parents of the next round of MEALE experiments performed monthly. When there were no great differences between the mutants and the initial parent on the growth rate in M9Y, mutants were transferred from M9Y to M9, and the previous steps were repeated until there were no great differences between the mutants and the initial parent on the growth rate in M9; thus, the expected mutants were achieved.

### 2.4. Measurement of β-Alanine and L-Aspartic Acid

The concentrations of β-alanine and L-aspartic acid in the culture broth were analyzed by high-performance liquid chromatography (HPLC) with the o-phthaldialdehyde derivatization (opa) method [18].

## 3. Results

### 3.1. Evolution of PPC Deficient E. Coli Strains

Due to the tremendous difference in energy regulation between the enzymes of PPC and PCK, PCK is more attractive in the anaerobic fermentation of β-alanine production. However, the enzymatic affinity of PPC is much higher than that of PCK. With the aim of discovering a mode with improved ATP and OAA availability for the production of β-alanine, the PPC-deficient strain B0016-090BB (B0016-082BB, *ppc*::FRT) was engineered for ALE experiments (Figure 1). ALE experiments were performed with different methods. The method of CALE was recruited in the first round. It takes approximately 1.5 months to reach an *OD*600 of 2. As the mutations accrue in the host, the evolution period becomes increasingly shorter. For one and a half years, approximately 800 generations, one clone was isolated and designated B0016-090BBS1 (B0016-090BB, ALE mutant).

For the purpose of accelerating the ALE experimental process, the MEALE method was applied, and experiments were carried out in quadruplicate. The PPC-deficient mutant B0016-090BB is lethal due to the breakoff of the metabolic flux at the phosphoenolpyruvate node. Nutrition contained in yeast extract, such as phosphoenolpyruvate and growth factors, can replenish the flux bottleneck and reinforce the growth of *E. coli*, which was designated marginal growth, with the assistance of yeast extract, based on the theoretic system of marginal effect to accelerate the growth rate. While the marginal growth rate was equal to that of the wild type, the mutants in M9Y acquired the desired genotypes and phenotypes and were then isolated and transfer-inoculated into M9 for further evolution [19]. It took half a year to achieve the mutants designated B0016-090BBS2 (B0016-090BB, ALE mutant), B0016-090BBS3 (B0016-090BB, ALE mutant), B0016-090BBS4 (B0016-090BB, ALE mutant), and B0016-090BBS5 (B0016-090BB, ALE mutant). Along with B0016-090BBS1, the adapted mutant pool was established.

**Figure 2:**
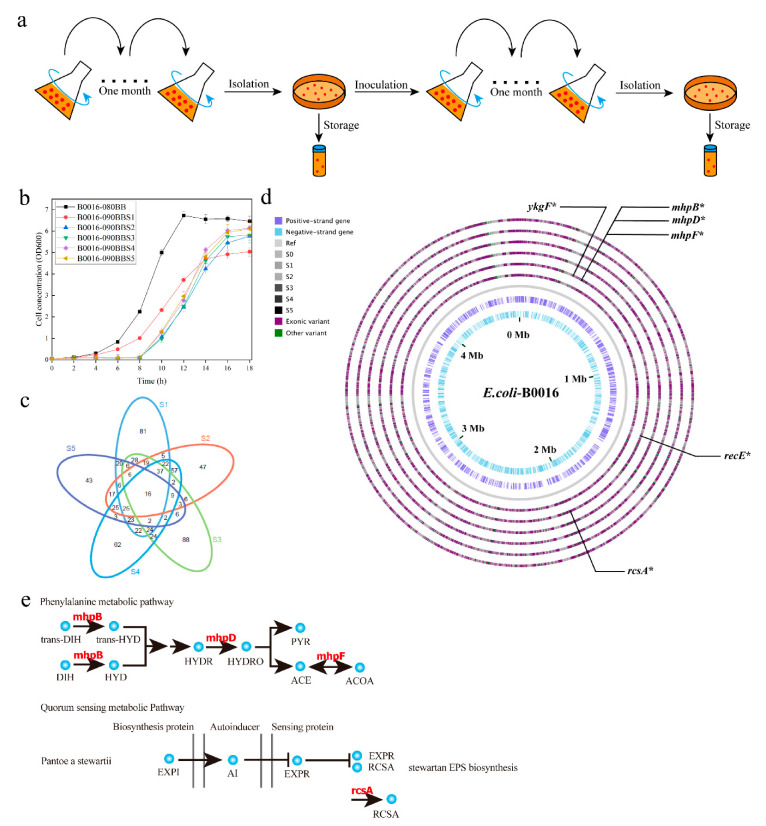
The method and results of ALE experiment. (**a**) The process of ALE experiment. (**b**) The growth curve of the adapted mutants. (**c**) Consistent mutations among the adapted mutants. (**d**) Genetic mutations of the mutants. Rings from outer to inner represent the genomic information of B0016-090BBS5, B0016-090BBS4, B0016-090BBS3, B0016-090BBS2, B0016-090BBS1, and B0016-090BB and the reference genome from the NCBI database. S0: B0016-090BB, S1: B0016-090BBS1, S2: B0016-090BBS2, S3: B0016-090BBS3, S4: B0016-090BBS4, S5: B0016-090BBS5. (**e**) The metabolic pathways associated with the genetic mutation library of the adapted mutant pool. DIH: 2.3-dihydroxycinnamate, HYD: trans- -hydroxy-6-oxononatrienedioate, HYDR: cis-2-hydroxypenta-2,4-dienoate, HYDRO: 4-hydroxy-2-oxopentanoate, PYR: pyruvate, ACE: acetaldehyde, ACOA: acetyl-CoA, EXPI: acyl homoserine lactone, AI: N-(3-oxohexanoyl)-L-homoserine lactone, EXPR: LuxR family transcriptional regulator, quorum-sensing system regulator ExpR, RCSA: LuxR family transcriptional regulator, capsular biosynthesis positive transcription factor.

In particular, ALE experiments should be suspended monthly to save frozen stocks of cells, allowing for characterization of both evolutionary intermediates and endpoint strains [15] (Figure 2a). The growth curves of the wild type and every adapted mutant in M9 medium are shown in Figure 2b, which indicated that mutations occurring in the ALE experiment can replenish the shortage of OAA caused by the deficit of PPC. Moreover, the application of a marginal effect can not only shorten the test cycle significantly, but also have a demonstrable effect on promoting and accumulating genetic mutations. Even though an obvious delay occurred since inoculation, the growth rate of all adapted mutants was equal to that of the wild type, which indicated the acquisition of desired mutants.

### 3.2. Establishment of the GML by Whole Genome Sequencing of the Adapted Mutant Pool

To investigate the physiological effects of the positive mutants responsible for the faster growth phenotype, whole genome sequencing of the wild type and the pool of adapted mutants were sequenced to establish GML, which is considered to be a powerful and reliable tool to predict and map compensatory metabolic flux distributions and enables the growth of lethal mutants as a potential result of evolutionary experiments [19]. As shown in Figure 2c, even though every round of the ALE experiment was performed independently, compared with the wild type, 16 types of consistent genetic mutations were discovered from all adapted mutants, which means that the GML consisting of these 16 genetic mutations was most likely to contribute to the faster growth phenotype. The details of the GML are shown in Figure 2d and Table 2. Focusing on the mutations of the coding frame, a frameshift mutation caused by the deletion mutation was performed on the *recE* mutant, which led to chaos of the whole reading frame, causing changes in protein properties. According to the database of Kyoto Encyclopedia of Genes and Genomes (KEGG, Bioinformatics Center, Kyoto University, Japan, https://www.kegg.jp/kegg/pathway.html (accessed on 15 February 2021)) and the database of the National Center for Biotechnology Information (NCBI, Bethesda, MD, USA, https://www.ncbi.nlm.nih.gov (accessed on 15 February 2021)), the enzyme encoded by *recE* possesses the activity of exonuclease VIII, which is related to the SOS response and DNA repair, functioning in homologous recombination by catalyzing DNA strand exchange reactions to repair DNA damage. Exonuclease VIII deficiency caused by frameshift mutations leads to a decline in DNA repair capacity, which enables and facilitates genetic mutations in ALE experiments.

Missense mutations caused by single-nucleotide variants (SNVs) responsible for the *mhpB* and *mhpD* and *mhpF* genetic mutations, and a frameshift mutation caused by an insertion was responsible for the *mhpF* mutant, which led to disruption of the whole reading frame, causing the changes in protein properties. According to the database of KEGG, the *mhpB* and *mhpD* and *mhpF* genes were all involved in the phenylalanine metabolic pathway (Figure 2e). As these genetic mutations contribute to the faster growth phenotype, we speculate that these mutations result in the accumulation of metabolic flux with the effect of facilitating growth and proliferation. With a missense mutation performed in *mhpF*, flux was blocked from acetaldehyde to acetyl-CoA, resulting in the accumulation of acetaldehyde and pyruvate. Pyruvate is an important metabolite related to growth and the synthesis of many other metabolites [20]. We proposed that the faster growth phenotype is presumably due to the accumulation of pyruvate, so the SNVs that occurred in *mhpB* and *mhpD* may enhance the activity of enzymes coded by *mhpB* and *mhpD*, channeling strong metabolic flux in the direction of pyruvate.

Compared with rational design, ALE provides a novel strategy for the accumulation of pyruvate by the phenylalanine metabolic pathway, and the accumulated pyruvate could be converted to PEP to provide a competent precursor for the reaction catalyzed by PCK, which can generate one stoichiometric ATP and fix one stoichiometric CO_2_ (Figure 1). It is difficult for rational design to regulate and redirect metabolic flux, and genetic manipulation may cause unforeseen effects on growth and the expression of necessary recombinant genes, as well as maintaining cofactors at optimal levels. The strategy of ALE could turn the integral carbon flux more subtle with far ranging global consequences of protein expression, yielding pleiotropic effects on cell growth and proliferation and resulting in the increased yield of target products [21]. This ATP generated evolution is also meaningful for the biosynthesis of ATP dependent products, such as succinate [22] and butanol [23], in the anaerobic fermentation.

Similar to the mutations involved in the phenylalanine metabolic pathway, mutations occurring in *ykgF* involved in the Embden–Meyerhof–Parnas (EMP) pathway were also related to pyruvate. According to the KEGG database, the *ykgF* gene is associated with the L-lactate dehydrogenase (LDH) complex protein LldF, which regulates the reaction from pyruvate to L-lactate catalyzed by LDH. To enhance the growth of mutants, we speculate that the genetic mutation occurring in the *ykgF* gene should weaken the activity of LDH to increase the concentration of pyruvate. Moreover, the reaction from pyruvate to L-lactate catalyzed by LDH is NADH dependent [24,25], and the production of L-lactate is a kind of byproduct that is not only toxic to the growth of *E. coli*, but also weakens the metabolic flux and reduces substrate conversion. The weakening of LDH can not only save reduction power to facilitate the NADH-dependent reaction from OAA to L-aspartate, but also increase the growth and proliferation of *E. coli*.

Genetic mutation also occurred in *rcsA*, which catalyzes the synthesis of stewartan exopolysaccharide (EPS). According to the KEGG database, *rcsA* is the transcriptional regulator of the LuxR family, which is related to quorum sensing. As shown in Figure 2e, *esaR* inhibited the expression of *rcsA* to control the biosynthesis of stewartan exopolysaccharide to regulate the cell membrane structure. Bacteria are a group of expansive interactive communities that exist in diverse multispecies environments [26]. Signaling molecules that exist in vivo and in vitro endow microorganisms with the ability to regulate a gamut of behavioral patterns [27] and the ability to respond to the environment by utilizing signaling molecules to standardize gene expression and establish the mode of communication among microbes. Signal molecules are synthesized intracellularly and diffuse passively or are actively released out of the cells [28]. The cell membrane plays an important role in the identification of signal molecules and the transduction of signals [29,30]. It was hypothesized that the genetic mutation occurring in *rcsA* might enhance quorum sensing by regulating the cell membrane structure and facilitating the communication and growth of *E. coli* [31,32].

### 3.3. Construction of the Cell Factory for the Biosynthesis of β-Alanine

**Figure 3:**
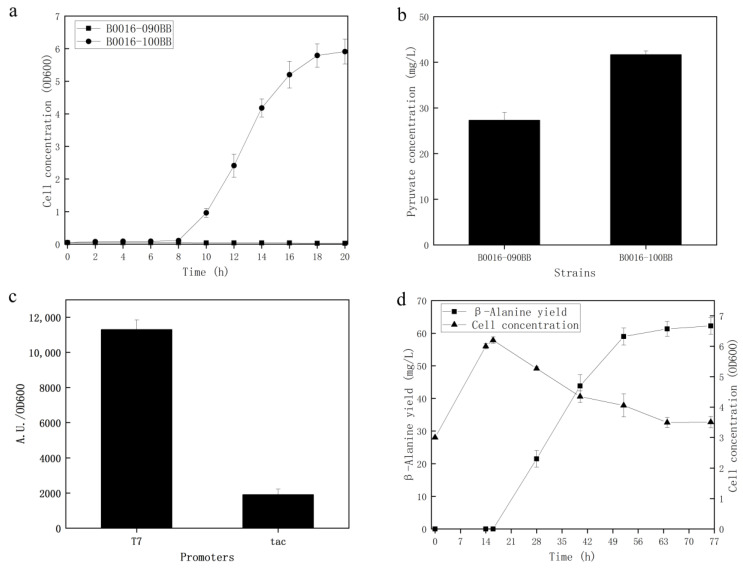
The profile of growth and production capability of the cell factory constructed with the guide of ALE. The growth curve (**a**) and the accumulation of pyruvate (**b**) were compared among B0016-090BB and B0016-100BB in M9 and M9Y media, respectively. The production capacity of B0016-200BB harboring the plasmid pET24a-*panD*-*AspDH* (**d**). Before bioconversion, *gfp* was applied as a reporter gene to compare the expression strength of tac and the T7 promoter (**c**) to optimize the expression cassette.

Microbial cell factories were engineered according to the GML (Table 2) and β-alanine biosynthesis pathways (Figure 1). Strain B0016-100BB (B0016-090BB, *recE*::FRT, *mhpF*::FRT, *ykgF*::FRT, *mhpB*:: *mhpB* *, *mhpD*:: *mhpD* *, *rcsA*:: *rcsA* *) evolved by introducing GML onto the chromosomes of B0016-090BB. As shown in Figure 3a, the chassis strain B0016-090BB was lethal in M9 medium, while the cell concentration of the evolved B0016-100BB reached an *OD*600 of 5.91 at 20 h, indicating that the consistent mutants revealed the anaplerotic strategy of OAA in B0016-090BB to enable the growth and proliferation of the PPC-deficient mutant. However, a long period of lag phase existed in the growth curve of B0016-100BB. Along with the low anaerobic growth rate of *E. coli* [33], it is not suitable for the subsequent production of β-alanine. To enhance the growth of *E. coli* and shorten the β-alanine biosynthesis fermentation period, M9Y medium was subsequently applied to replenish growth factors [34]. To test the hypothesis that the consistent genetic mutations that occurred in ALE could replenish pyruvate to contribute to the faster growth phenotype, the concentration of pyruvate was compared among B0016-090BB and B0016-100BB cells cultured in M9Y medium. As shown in Figure 3b, pyruvate synthesized by B0016-100BB was 1.5 times that synthesized by B0016-090BB. This result indicated that with the guidance of ALE, compared with the wild-type B0016-090BB, the pyruvate accumulation capacity of B0016-100BB was improved, which could be considered evidence to support our conjecture.

**Figure 4:**
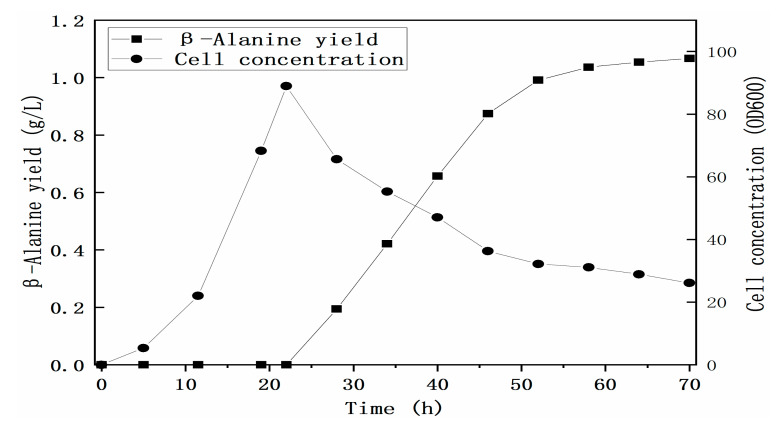
Profiles of growth and β-alanine production using strain B0016-200BB/pET24a-*panD*-*AspDH* in a 5-L bioreactor. During the fermentation of β-alanine production, genes were induced for expression with the addition of IPTG at 15 h, and oxygen-limited fermentation was conducted at 22 h.

To build and reinforce the metabolic pathway of the β-alanine biosynthesis pathway, heterologous genes were cloned and overexpressed. According to the KEGG database and the BRENDA enzyme database (Institute of Biochemistry, Universität zu Köln, Germany, https://www.brenda-enzymes.org/ (accessed on 15 February 2021)), the *AspDH* gene from *Klebsiella pneumonia*, a facultative anaerobic strain, can express the AspDH enzyme and catalyze the reaction from OAA to L-aspartate, and the *panD* gene from *Corynebacterium glutamicum* can express the ADC enzyme and catalyze the reaction from L-aspartate to β-alanine. Considering the activity of anaerobic fermentation, these two genes were assembled into an operon in the modified vector pET24a-tac under the control of a weaker tac promoter, resulting in the plasmid pET24a-*panD*-*AspDH* (Figure 1). To block the aerobic metabolic pathway of β-alanine biosynthesis, B0016-200BB (B0016-100BB, *aspA*::FRT) was engineered to block the flux from fumaric acid to L-aspartate by knocking out the *aspA* gene. *E. coli* B0016-200BB harboring the plasmid pET24a-*panD*-*AspDH* was selected for the production of β-alanine. As shown in Figure 3d, the production of β-alanine reached 62.23 mg/L at 76 h, and L-aspartate could not be detected in anaerobic fermentation, suggesting that even though the T7 promoter was replaced by a weaker tac promoter (Figure 3c), the node from L-aspartate to β-alanine was no longer the bottleneck pathway. The application of weaker promoters can mitigate the metabolic burden caused by overexpression and balance endogenous metabolic pathways more efficiently. The production of the starting strain B0016-082B (B0016-082BB, *aspA*::FRT) harboring the plasmid pET24a-*panD*-*AspDH* was also detected; however, only 0.79 mg/L β-alanine was generated. It indicated that the production efficiency of β-alanine in the engineered cell was significantly improved. An interesting phenomenon is shown in Figure 3d; even though IPTG was added at 14 h to trigger an OFF to ON state for gene expression, no β-alanine was generated in the ensuing aerobic fermentation. β-Alanine biosynthesis was in progress until anaerobic fermentation was performed. A similar phenomenon is shown in Figure 4. No β-alanine was generated in the intermediate period between IPTG addition and anaerobic fermentation, and no L-aspartate could be detected in anaerobic fermentation, while the production of β-alanine reached 1.07 g/L at 70 h in fed-batch fermentation. We proposed that the mechanism may occur for two reasons. The first is the electron transport chain, which is a series of complexes that transfer electrons from electron donors to electron acceptors via redox (both reduction and oxidation occurring simultaneously) reactions and couple this electron transfer with the transfer of protons (H^+^ ions) across a membrane [35]. Molecular oxygen (O_2_) plays a role as a sufficient and powerful electron acceptor. However, the reaction from OAA to L-aspartate is an NADH-dependent process, as molecular oxygen is a high-energy oxidizing agent [36]; therefore, it is competitive in both hydrogen transfer reactions and electron transfer reactions [37] and leads to the lack of NADH in the reaction from OAA to L-aspartate, which may be summarized as competitive inhibition. The second reason is related to energy. Both cell growth and metabolite synthesis are energy-consuming processes. Endogenous metabolic pathways are naturally channeled in the direction of energy-generating pathways. The TCA cycle is known to be the main energy source that competes OAA with the reaction catalyzed by AspDH to weaken the anaerobic biosynthesis of β-alanine, which may also be summarized as competitive inhibition. Based on the competition of cofactors and precursors, only if the limited supply of molecular oxygen triggers an OFF state in aerobic fermentation to abolish the activity of the TCA cycle can metabolic flux no longer channel to the direction of β-alanine biosynthesis from the direction of the TCA cycle. Along with the utilization of anaerobic fermentation, the main energy source would turn from oxidative phosphorylation, the TCA cycle, to substrate-level phosphorylation, the node from PEP to OAA catalyzed by PCK. Moreover, sufficient NADH is available from glycerol glycolysis to trigger the reaction from OAA to β-alanine biosynthesis.

## 4. Conclusions

A novel mode of energy generation and CO_2_ conversion strategy along with β-alanine biosynthesis was engineered with the guide of adaptive laboratory evolution. Based on the genetic mutation library established, and based on the consistent genetic mutation of adapted mutants, pyruvate can be replenished from the phenylalanine metabolic pathway and the EMP pathway, and sufficient pyruvate can be converted to PEP to provide a competent precursor for the reaction catalyzed by PCK, which can generate one stoichiometric ATP. Moreover, the energy generation pathway catalyzed by PCK can convert CO_2_ into metabolites related to the biosynthesis of β-alanine, making the β-alanine biosynthesis process much more profitable. As a replenishment strategy of rational design, which requires sufficient knowledge of metabolic mechanisms and tremendous workload, ALE provided a novel strategy to fine-tune the global metabolism. Alternations in far ranging metabolic routes would yield pleiotropic effects on cell growth and result in increased yield of the target products. This novel strategy should be widely distributed to solve the great challenge of the energy supply existing in anaerobic fermentation to enable the production of target metabolites. The strategies of energy regulation and carbon partitioning are meaningful for the rechanneling of intracellular metabolites and the production of C4 platform chemicals.

## Figures and Tables

**Table 1:** *E. coli* strains and plasmids used in this study.

Strains	Relevant Characteristics	Source or Reference
B0016-082BB	B0016-080BB, *panD*:: Cg*panD*	Lab stock
B0016-082B	B0016-082BB, *aspA*::FRT	This study
B0016-090BB	B0016-082BB, *ppc*::FRT	This study
B0016-090BBS1	B0016-090BB, ALE mutant	This study
B0016-090BBS2	B0016-090BB, ALE mutant	This study
B0016-090BBS3	B0016-090BB, ALE mutant	This study
B0016-090BBS4	B0016-090BB, ALE mutant	This study
B0016-090BBS5	B0016-090BB, ALE mutant	This study
B0016-100BB	B0016-090BB, *recE*::FRT, *mhpF*::FRT, *ykgF*::FRT, *mhpB*:: *mhpB* *, *mhpD*:: *mhpD* *, *rcsA*:: *rcsA* *	This study
B0016-200BB	B0016-100BB, *aspA*::FRT	This study
B0016-082B/pET24a-*panD*-*AspDH*	B0016-082B with pPL451-*panD*-*AspDH* plasmid	This study
B0016-200BB/pET24a-*panD*-*AspDH*	B0016-200BB with pPL451-*panD*-*AspDH* plasmid	This study

* Genes containing genetic mutations are listed in the genetic mutation library.

**Table 2:** The genetic mutation library of the adapted mutant pool.

Genes	Mutations	Amino Acid Mutations
*recE*	1737–1737 del ^a^, 1739–1741 del	Frameshift mutation
*mhpB*	A107C ^b^	E36A ^d^
*mhpD*	T278C	I93T
*mhpF*	T179C, G189C, 76–77 ins GC ^c^, 116–117 del, 120–121 ins AA	Frameshift mutation
*ykgF*	A19G, T27A,33–35 del, 37–38 ins T, 39–40 ins GT, C94G,	Frameshift mutation
*rcsA*	T209A	L70H

^a^ Acid bases deletion, ^b^ Acid bases substitution, ^c^ Acid bases insert, ^d^ Amino acid substitution.

## Data Availability

The data presented in this study are available on request from the corresponding author.

## Supplementary Materials

The following are available online at https://www.mdpi.com/2076-2607/9/3/600/s1: Table S1: Plasmids used in this study, Table S2: Primers used in this study.
