# Supplementary material for: Novel Mode Engineering for β-Alanine Production in Escherichia coli with the Guide of Adaptive Laboratory Evolution
Source: Microorganisms. 2021 Mar 15;9(3):600. doi: 10.3390/microorganisms9030600 (PMC8000549; doi:10.3390/microorganisms9030600)
Supplement: Supplementary file 1 [file microorganisms-09-00600-s001.pdf]

Table S1. Plasmids used in this study.

Plasmids	Relevant characteristics	Source or reference
pET24a	Km^R,T7 promoter, T7 terminator	Lab stock
pET24a-tac	T7 promoter was replaced with tac promoter, T7 tag and 6×His tag was deleted	This study
pET24a-*panD*	the T7 tag and 6×His tag of pET24a-tac was replaced with the *panD* gene from *Corynebacterium glutamicum*	This study
pET24a-*AspDH*	the T7 tag and 6×His tag of pET24a-tac was replaced with the *AspDH* gene from *Klebsiella pneumoniae*	This study
pUC19	Ampr	Lab stock
pUC19-FRT-Kan-FRT	the cassette of FRT-Kan-FRT was amplified from pKD13 plasmid and cloned onto the plasmid of pUC19	This study
pUC19-FRT-Kan-FRT- *mhpB*	*mhpB* gene was amplified and cloned onto the plasmid of pUC19-FRT-Kan-FRT	This study
pUC19-FRT-Kan-FRT-*mhpB*[*]	*mhpB* gene containing all genic mutations listed in the genic mutation library	This study
pUC19-FRT-Kan-FRT-*mhpD*	*mhpD* gene was amplified and cloned onto the plasmid of pUC19-FRT-Kan-FRT	This study
pUC19-FRT-kan-FRT- *mhpD* [*]	*mhpD* gene containing all genic mutations listed in the genic mutation library	This study
pUC19-FRT-Kan-FRT- *rcsA*	*rcsA* gene was amplified and cloned onto the plasmid of pUC19-FRT-Kan-FRT	This study
pUC19-FRT-kan-FRT- *rcsA* [*]	*rcsA* gene containing all genic mutations listed in the genic mutation library	This study
pET24a-*panD*-*AspDH*	pET24a-tac containing the *panD* gene from *Corynebacterium glutamicum* and the *AspDH* gene from *Klebsiella pneumoniae*	This study
pET24a-*gfp*	pET24a containing *gfp* gene	This study
pET24a-tac-*gfp*	pET24a-tac containing *gfp* gene	This study
pKD46	Ampr, $\gamma\beta$exo (red recombinase), Temperature-conditional replicon	CGSC[a][40]
pKD13	Amp^r, Kan^r, FRT-Kan-FRT cassette	CGSC[a][40]
pCP20	Amp^r, FLP, Temperature-conditional replicon	CGSC[a][40]

[a] Single-nucleotide variant. [b] Deletion mutation. [c] Insertion mutation. [d] Amino acid mutations.

Table S2. Primers used in this study.

Primers	Sequence (5′-3′) restriction italic/underlined	Restriction sites
ppc-pKD13F	GAAGGATACAGGGCTATCAAACGATAAG ATGGGGTGTCTGGGGTAATATGGTGTAGG CTGGAGCTGCTTC	
ppc-pKD13R	AAAGCACGAGGGTTTGCAGAAGAGGAA GATTAGCCGGTATTACGCATACCATTCCG GGGATCCGTCGACC	

Y*ppc*F	GTAGAGCAGTGCCCAACCCAGG
Y*ppc*R	TTTGCTGAAGCGATTTCGCAG
recE-pKD13F	GCAGATCGGCTTTTGCGATTGGTGGTTGC TTAGTCATTTGCATATTCCTTGTGTAGGCT GGAGCTGCTTC
recE-pKD13R	ATTCACTGAACAAAACGAATTTTAATCTG AGTTGAGGTTAAAAAACAATGATTCCGG GGATCCGTCGACC
Y*recE*F	CCGAGCTGTGAACACTGTACGAT
Y*recE*R	CACTGTGGTTGATATCGATGGTCC
mhpF-pKD13F	TCAGTTGCTGCGACATTTTCAAGCGCAGC CCCAAAAGGAAGTCTGTCATGGTGTAGG CTGGAGCTGCTTC
mhpF -pKD13R	GACGTCCGAGATATAAAGTTTTTTACCGT TCATGCCGCTTCTCCTGCCTTATTCCGGGG ATCCGTCGACC
Y *mhpF*	CAAAATGGCCAGTCTGGGTGAAC
Y *mhpF*R	CACTTCAATCGAATCCACGCG
ykgF-pKD13F	AGTGATGCATATTGCTGAAGTGTTGATGA GCCGCTGAGGATATAAAGATGGTGTAGG CTGGAGCTGCTTC
ykgF-pKD13R	GAGCAACGTTATTCAAAAATTCGCCTCGA TTATCCATTCTTTTTCTCCTGATTCCGGGG ATCCGTCGACC
Y*ykgF*F	TCAAAATGGCCGAAATATCCG
Y*ykgF*R	AACATAACATCGCTGGCAAACTG
mhpB –pUC19F	ATGCACGCTTATCTTCACTGTCTTTCCCAC TCGCCGCTGGTGGGGTATGTGTGTAGGCT GGAGCTGCTTC
mhpB –pUC19R	TCAGTTCTCTGTTCTGGCGCTTAACGAGC CAAATCCGGCAATCCACTCCG
Y*mhpB*F	ATTCATACCGCACAGGATAACCAC
Y*mhpB*R	AACCATGCAGCAGGACAACG
mhpD –pUC19F	ATGACGAAGCATACTCTTGAGCAACTGG CGGCGGATTTACGCCGCGCCGCGTGTAG GCTGGAGCTGCTTC
mhpD –pUC19R	TCATGACAGACTTCCTTTTGGGGCTGCGC TTGAAAATGTCGCAGCAACTG
Y*mhpD*F	GCCTGGAAGCTAATCCGAAACA
Y*mhpD*R	TCAGGCATGTTCATCAGTCCG
aspA-pKD13F	GCCTTTTTTATTTGTACTACCCTGTACGAT TACTGTTCGCTTTCATCAGTGTGTAGGCTG GAGCTGCTTC
aspA-pKD13R	CTGTGTGTTTAAAGCAAATCATTGGCAGC TTGAAAAAGAAGGTTCACATGATTCCGG GGATCCGTCGACC
Y*aspA*F	CCAATAGCGGCAAGAACCAG
Y*aspA*R	CGAATCGCGTTTAGCTTATATTGTG
rcsA –pUC19F	ATGTCAACGATTATTATGGATTTATGTAG TTACACCCGACTAGGTTTAACGTGTAGGC TGGAGCTGCTTC

rcsA–pUC19R	TTAGCGCATGTTGACAAAAATACCATTAGTCACATTATCCGTCAGTCGGA	
Y*rcsA*F	AAGCTCACTCACATATCGCAACAT	
Y*rcsA*R	GGTACTGGCAGTTGAGGAGTTTAGTG	
HindIII-FRT-Kan-FRTF	TTT*AAGCTT*[a]GTGTAGGCTGGAGCTGCTTC	HindIII[a]
HindIII-FRT-Kan-FRTR	TTT*AAGCTT*[a]ATTCCGGGGATCCGTCGACC	HindIII[a]
BamHI- *mhpB*F	TTT*GGATCC*[a]ATGCACGCTTATCTTCACTGTCTTTC	BamHI[a]
EcoRI- *mhpB*R	TTT*GAATTC*[a]TCAGTTCTCTGTTCTGGCGCTTAA	EcoRI[a]
mhpB[*]F	ATTGCCAGCGCCCGCGCGCGTATTGCGG	
mhpB[*]R	GAGAATGCCGCAATACGCGCGCGGGCGCT	
BamHI- *mhpD*F	TTT*GGATCC*[a]ATGACGAAGCATACTCTTGAGCAACT	BamHI[a]
EcoRI- *mhpD*R	TTT*GAATTC*[a]TCATGACAGACTTCCTTTTGGGG	EcoRI[a]
mhpD[*]F	TGTGTTATGGCGATAACGAAACCATTCCTTTTT	
mhpD[*]R	GTTGCAGAACACGGGAAAAAGGAATGGTTTCGTTATC	
BamHI- *rcsA*F	TTT*GGATCC*[a]ATGTCAACGATTATTATGGATTTATGTAGTTAC	BamHI[a]
EcoRI- *rcsA*R	TTT*GAATTC*[a]TTAGCGCATGTTGACAAAAATACC	EcoRI[a]
rcsA[*]F	TCTAACAGTCAGCGTATCAAGCACATCATTAATCAA	
rcsA[*]R	TATTGGGATGTTGATTAATGATGTGCTTGATACGC	
pET24a- tacF	TCCGCTCACAATTCCCATTATACGAGCCGATGATTAATTGTCAAATTTCGCGGGATCGAGATCTC	
pET24a- tacR	TCGGCTCGTATAATGGGAATTGTGAGCGGATAACAATTCC	
panD- pET24aF	AATGGTGCGCAGCATATGTATATCTCCTTCTTAAAGTTAAACAAAATTATTTCTAGA	
panD- pET24aR	AGCCGCAGCATCTAACCCGAAAGGAAGCTGAGTTGG	
pET24a- *panD*F	GAAGGAGATATACATATGCTGCGCACCATTCTGG	
pET24a- *panD*R	CAGCTTCCTTTCGGGTTAGATGCTGCGGCTGGTCAG	
AspDH- pET24aF	CACTTTCTTCATCATATGTATATCTCCTTCTTAAAGTTAAACAAAATTATTTCTAGA	
AspDH- pET24aR	CGTGAACTGGCCTAACCCGAAAGGAAGCTGAGTTGG	
pET24a- *AspDH*F	GAAGGAGATATACATATGATGAAGAAAGTGATGCTGATCG	
pET24a- *AspDH*R	CAGCTTCCTTTCGGGTTAGGCCAGTTCACGGCAC	

panD- pET24a-*AspDH*F	GCGGGATCGAGATCTGAAGATCGGGCTCGCCACTT	
panD- pET24a-*AspDH*R	TTGAGGGGTTTTTTGCGGTGATGTCGGCGATATAGGC	
pET24a- *AspDH*-*panD*F	GCGAGCCCGATCTTCAGATCTCGATCCCGCGAAAT	
pET24a- *AspDH*-*panD*R	TCGCCGACATCACCGCAAAAAACCCCTCAAGACCCG	
gfp-BamHIF	TTT*GGATCC*[a]ATGAGTAAAGGAGAAGAACTTTTCACTGG	BamHI[a]
gfp-EcoRIR	TTT*GAATTC*[a]TTATTTGTATAGTTCATCCATGCCATGT	EcoRI[a]
Ypet24aF	ATCATGCCATACCGCGAAAGG	
Ypet24aR	GCTTAATGCGCCGCTACAGG	

[a] Restriction sites with corresponding restriction enzymes. * Primer of site-directed mutagenesis.
